# The Lifestyle Profile of Individuals with Cardiovascular and Endocrine Diseases in Cyprus: A Hierarchical, Classification Analysis

**DOI:** 10.3390/nu14081559

**Published:** 2022-04-08

**Authors:** Maria Kyprianidou, Demosthenes Panagiotakos, Konstantinos C. Makris, Maria Kambanaros, Costas A. Christophi, Konstantinos Giannakou

**Affiliations:** 1 Cyprus International Institute for Environmental and Public Health, Cyprus University of Technology, Limassol 3036, Cyprus; kyprianidou.maria.ky@gmail.com (M.K.); konstantinos.makris@cut.ac.cy (K.C.M.); costas.christophi@cut.ac.cy (C.A.C.); 2 Department of Health Sciences, School of Sciences, European University Cyprus, Nicosia 1516, Cyprus; 3 Department of Nutrition and Dietetics, School of Health Sciences and Education, Harokopio University, 17676 Athens, Greece; dbpanag@hua.gr; 4 Department of Allied Health and Human Performance, University of South Australia, Adelaide, SA 5001, Australia; maria.kambanaros@cut.ac.cy

**Keywords:** cardiovascular disease, endocrine disease, lifestyle, Cyprus, classification analysis

## Abstract

The study aims to explore the lifestyle profile of adult individuals with cardiovascular and endocrine diseases in Cyprus. Age and sex-specific analyses were applied. A representative sample of the general adult population was recruited during 2018–2019 using stratified sampling among the five government-controlled municipalities of the Republic of Cyprus. Data on Mediterranean diet adherence, quality of sleep, smoking status, physical activity, Body Mass Index, and the presence of cardiovascular and endocrine diseases were collected using a validated questionnaire. Diseases were classified according to the International Classification of Diseases, Tenth Revision (ICD-10). A total of 1140 men and women over 18 years old (range: 18–94) participated in the study. The prevalence of cardiovascular and endocrine diseases among the adult general population of Cyprus was 24.8% and 17.2%, respectively, with a higher prevalence of cardiovascular diseases in men, and a higher prevalence of endocrine diseases in women. Among individuals with cardiovascular disease, 23.3% were aged between 18–44 years old, while the corresponding percentage among endocrine disease individuals was 48%. The prevalence of smoking, physical activity, a low adherence to the Mediterranean diet, poor quality of sleep and obesity among the study population was 35.5%, 48.0%, 32.9%, 39.0% and 13.6%, respectively. Individuals with cardiovascular and endocrine diseases were characterized by poor quality of sleep, inadequate physical activity, and a higher BMI. This is the first study in Cyprus exploring the profile of individuals with cardiovascular and endocrine diseases in Cyprus. Health promotion and educational programs focusing on the importance of sleep quality, healthier dietary habits, physical activity, and lower BMIs among people with cardiovascular and endocrine diseases should be developed.

## 1. Introduction

Chronic diseases are a major threat to economies and societies [1]. Recent studies have reported that the most prevalent chronic diseases are the diseases of the cardiovascular and endocrine systems [1,2,3,4,5,6,7,8], whilst cardiovascular diseases have been reported as the most frequent cause of death worldwide [7,8] with an estimated 17.9 million deaths per year [9]. Chronic diseases, including cardiovascular diseases and diabetes, are responsible for almost 50% of the global burden of disease and 59% of the annual deaths [10]. 

In 2021, the World Health Organization (WHO) reported that tobacco use, inactivity, problematic alcohol use and poor diet raise significantly, the likelihood of dying from a non-communicable illness [11] and are associated with the incidence of various chronic diseases [12,13,14,15]. Lifestyle habits, including diet, physical activity, smoking and sleep, have been changing over the years, primarily due to urbanization [16] and the adoption of a Western lifestyle [17,18], especially among developing countries, and have been associated with increased morbidity and mortality [19,20,21,22]. Poor quality of sleep is another lifestyle factor that has been associated with a higher risk for cardio-metabolic diseases [23], diseases of the endocrine system [24], such as diabetes, hypertension [25,26], and multimorbidity [27]. 

In Cyprus, the major causes of death are ischemic heart disease, stroke and diabetes, with the diabetes mortality rate being the highest in the European Union (EU) [15]. It has been reported that 20% of the total mortality in 2019 was due to dietary risks, including low fruit and vegetable consumption and high consumption of sugar and salt, while the corresponding percentage in Europe was 18% [28]. In addition, the percentage of daily smokers among Cypriot adults in 2014 was 26% (38% of males and 14% of females), which is among the highest in all European countries [28] and higher than the EU average [15]. Of note, Cyprus has one of the highest life expectancies among European countries, 82.2 years old (83.1 years for women and 78.4 years for men), and this is expected to increase over the decades [15,29]. 

Unfortunately, there is a lack of epidemiological data regarding the burden of chronic diseases and the lifestyle habits of the population of Cyprus. WHO notes that the investment of society in the management of chronic diseases is crucial, and an important way to do that is through prevention, by controlling the risk factors associated with these diseases [11]. Given the lack of data and the fact that chronic diseases are the main source of morbidity and mortality, it is important to examine the lifestyle factors associated with these diseases. Thus, the aim of this study is to analyze the distribution of lifestyle factors among the adult general population of the Republic of Cyprus, overall and in individuals with cardiovascular and endocrine diseases, and to generate the profile of adult individuals with cardiovascular and endocrine diseases.

## 2. Materials and Methods

### 2.1. Study Design

This was a cross-sectional study.

### 2.2. Setting

The study population included both men and women aged 18 years old and older who resided in the five government-controlled municipalities of the Republic of Cyprus (Nicosia, Limassol, Larnaca, Paphos and Ammochostos). Individuals living in nursing homes or those institutionalized were excluded. Data collection took place from May 2018 to June 2019.

### 2.3. Sampling

We applied a stratified sampling method to ensure that the study sample matched the population of Cyprus in three key demographic characteristics (age, sex and residency). We used the latest available census data (2011), and we divided the referent population into the five municipalities of Cyprus. We further stratified the population according to the type of residence (urban and rural, as provided by the National Bureau of Statistics), gender (men and women), and age group (18–24, 25–44, 45–64, 65+ years old). Recruitment took place via face-to-face interviews by trained investigators in public places and in houses throughout Cyprus. A representative sample was collected (Nicosia (43% of the total Cypriot population), Limassol (27%), Larnaca (15%), Paphos (10%), and Ammochostos (5%)) since our study sample was similar to the general population of Cyprus with respect to region, age and gender (all *p*-values > 0.05).

### 2.4. Participants’ Characteristics

A standardized questionnaire was used for data collection. The questionnaire included different sections on sociodemographic characteristics, medical history, dietary habits, as well as information regarding the quality of sleep, smoking and physical activity.

#### 2.4.1. Sociodemographic Characteristics

Age was reported in years and sex was recorded as men or women. Age was classified into four categories based on the age categorization of the Statistical Service of Cyprus [28]: 18–24 years old, 25–44 years old, 45–64 years old and more than 65 years old. Educational level was classified in three categories as commonly used in Cyprus and similar to a study on the Greek population [30], namely: (i) primary education (participants who completed only primary school—<7 years of schooling); (ii) secondary education (participants who completed middle or high school—7–12 years of schooling); (iii) higher education (participants who have a university degree—>12 years of schooling) [30]. Job status was recorded as a private or state employee, farmer, student, unemployed, freelance, housewife or retired. 

#### 2.4.2. Anthropometric Characteristics

Weight and height were provided in kilograms and in meters, respectively. BMI was calculated as weight (kilograms) divided by height (meters squared). Obesity was defined as BMI > 29.9 kg/m^2^, overweight as BMI 25–29.9 kg/m^2^, normal as BMI 18.5–24.9 kg/m^2^, and underweight as BMI < 18.5 kg/m^2^, according to the WHO classification. 

#### 2.4.3. Smoking Habits

Smoking was evaluated using a question about current smoking status (i.e., current smoker, non-smoker), if they ever smoked regularly and their age when smoking started.

#### 2.4.4. Physical Activity Assessment

The physical activity questionnaire comprised of questions about physical activity habits, including the type of exercise and duration of exercise. The type of exercise was categorized as football, volleyball, basket, swimming, martial arts, gym, track, jogging, and cycling, and there was an option to report another type of exercise. Duration of exercise per week was given as less than one hour, 1–3 h, 3–6 h, 6–9 h, and more than 9 h.

#### 2.4.5. Dietary Habits Assessment

The section on dietary habits included a detailed validated semi-quantitative food frequency questionnaire (FFQ) [31]. The validation of the FFQ was based on participants from Athens, Greece [31] and the questionnaire has been found to provide an acceptable assessment of long-term dietary intakes in the general population in various countries and regions. Moreover, it is known that the Cypriot population has similar characteristics to the Greek population, including dietary habits, as both Greeks and Cypriots share common foods, cooking practices, and eating behaviors. The FFQ included specific local food groups, such as Cyprus local cheese (halloumi) and other local products.

The FFQ included the consumption of 11 food groups: non-refined cereals, fruits, vegetables, legumes, potatoes, fish, meat and meat products, poultry, full-fat dairy products, as well as olive oil and alcohol intake. Adherence to the Mediterranean diet was evaluated using the MedDietScore [32]. The MedDietScore has a score range of 0–55, with higher values indicating greater adherence to the Mediterranean diet. For the consumption of items presumed to be close to this, pattern scores were assigned as 0, 1, 2, 3, 4 and 5 when a participant reported no consumption, rare, frequent, very frequent, weekly, and daily consumption, respectively. For the consumption of foods presumed to be away from this, pattern scores were assigned in the reverse order.

#### 2.4.6. Quality of Sleep Assessment

Quality of sleep was assessed using the Pittsburgh Sleep Quality Index (PSQI) questionnaire in Greek [33] as kindly provided by the University of Pittsburgh. The PSQI consists of 19 self-rated questions (an additional 5 questions that are rated by the bed-partner or roommate, which are used for clinical information only and are not part of the calculation of the PSQI score, were not used in the study). The 19 self-rated questions assess a wide variety of factors relating to sleep quality, including estimates of sleep duration and latency, and of the frequency and severity of specific sleep-related problems. These 19 items are grouped into 7 components (subjective sleep quality, sleep latency, sleep duration, habitual sleep efficiency, sleep disturbances, use of sleeping medications, and daytime dysfunction), each scored on a 0–3 scale. The seven component scores are then summed to yield a global PSQI score, which has a maximum score of 21, with higher scores indicating worse sleep quality.

#### 2.4.7. Participants’ Medical History

The questionnaire’s medical history section included 47 chronic diseases of all the human systems (i.e., cardiovascular, digestive/excretory, endocrine, immune, nervous, renal/urinary, reproductive, respiratory, and skeletal/muscular systems, neoplasms) which were coded according to the international classification of diseases (ICD-10). This section was completed by the researchers using the question “Have you ever been diagnosed by a physician with any of the following chronic diseases? Choose all that apply”. The categorization of the chronic diseases was as follows: diseases of the cardiovascular system included hypertension (I10), hyperlipidemia (E78.5), angina (I20.9), coronary heart disease (I25.1), atrial fibrillation (I48.91) and heart failure (I50), while diseases of the endocrine system included: type 1 diabetes mellitus (E10), type 2 diabetes mellitus (E11), thyroid diseases (E02, E03.8, E03.9, E05.90, E07.9), and polycystic ovarian syndrome (E28.2).

### 2.5. Ethics Approval

The study was approved by the Cyprus National Bioethics Committee (CNBC) (ΕΕΒΚ ΕΠ 2018.01.123). Details about the study’s aims and procedures were provided to each potential participant before joining the study. Specifically, after explaining the purpose of the study to the participants, the researchers would point out that the study has been approved by the CNBC and that their participation would be anonymous as well as that they could stop participating at any time they wanted to. The researchers would then ask for the participants’ consent to participate, and they would provide the questionnaire of the study for completion. The researchers would also explain to the study participants that the questionnaire is self-reported, except for the medical history section, which would be performed with a face-to-face interview.

### 2.6. Statistical Analysis

The distribution of the continuous variables was examined using the Shapiro–Wilk normality test. Continuous variables with normal distributions were presented as mean ± standard deviation (SD) and continuous measures with skewed distributions as median (q_1_:1st quartile, q_3_:3rd quartile). Categorical variables were presented as absolute (n) and relative (%) frequency. The chi-square test of independence was used to evaluate any association between the categories of cardiovascular and endocrine diseases and the characteristics of the participants. The Student’s *t*-test and the Wilcoxon rank-sum test were used for the comparison of the groups with and without cardiovascular disease and with and without endocrine disease in terms of their average level of the continuous baseline characteristics with normal distribution, and for continuous characteristics with skewed distributions, respectively.

We have calculated and used the tertiles of the actual dataset dividing the sample into equal size subgroups. The tertiles of adherence to the Mediterranean diet were defined as follows: low adherence (score equal or smaller than 13), moderate adherence (score 14–18), and high adherence (score equal or greater than 19). Quality of sleep was defined as follows: good quality of sleep (score equal to or smaller than 5) and poor quality of sleep (score greater than 5). Bubble graphs were constructed to present the most common combinations of chronic diseases of cardiovascular and endocrine human systems.

Discriminant classification analysis and logistic regression are multivariate statistical methods that can be applied for the estimation of the associations between various variables and a categorical outcome [34]. However, discriminant analysis is used to evaluate the set of variables that discriminate between two or more two naturally occurring groups and to classify an observation into these known groups since it produces a discriminate score for each group with different classification abilities [34]. The study aims to generate the profile of adult individuals with cardiovascular and endocrine diseases overall, but also separately for each gender and each age group. Hence, we performed a discriminant classification analysis, with the calculation of Wilk’s lambda (the closer to 1, the better the discriminating ability) and Fisher’s classification function coefficients to evaluate the patterns of characteristics of people with cardiovascular and endocrine disorders overall, by gender, and by the four age groups of the study population (18–24, 25–44, 45–64 and 65+ years old). All statistical tests performed were two-sided, with the statistical significance level set at α = 0.05. Statistical analysis was conducted using STATA 14.0 (Stata Corp, College Station, TX, USA) and R statistical software.

## 3. Results

### 3.1. Participants’ Characteristics

There were a total of 1140 participants, among whom 642 (56.4%) were women (Table 1). The mean age of the participants was 41 ± 17 years (15% (*n* = 167) aged 18–24 years old, 46% (*n* = 524) aged 25–44 years old, 27% (*n* = 314) aged 45–64 years old, and 12% (*n* = 135) aged 65 years and older). Most of the participants were from the capital of Cyprus, Nicosia (*n* = 493, 43.3%), residents of urban regions (*n* = 864, 76.3%), 64% (*n* = 729) had completed a higher education, and 40% (*n* = 432) were private employees. More details about the demographics, socioeconomic characteristics of the participants are presented in Table 1.

### 3.2. Participants’ Characteristics by Cardiovascular and Endocrine Diseases

The prevalence of cardiovascular diseases and endocrine diseases was 24.8% and 17.2%, respectively. Among individuals with cardiovascular disease, the most prevalent chronic diseases were hyperlipidemia (69.8%), hypertension (51.6%), followed by heart failure (6.0%), atrial fibrillation (4.2%), angina (3.2%) and coronary heart disease (3.2%). In addition, among individuals with endocrine diseases, the most prevalent chronic disease was thyroid diseases (49.0%), followed by polycystic ovarian syndrome (35.9%), type 2 diabetes (13.6%) and type 1 diabetes (13.1%). 

Individuals with cardiovascular and endocrine diseases were significantly older than the participants without cardiovascular and endocrine diseases (55 ± 16 vs. 36 ± 14 years old and 47 ± 18 vs. 40 ± 16 years old, respectively, *p* < 0.05) (Table 1). The proportion of participants with cardiovascular diseases among those aged 18–24 years old, 25–44 years old, 45–64 years old, and more than 65 years old were 3.6%, 11.5%, 40.1% and 67.4%, respectively (*p* < 0.01). Similarly, the prevalence of endocrine diseases in the four age groups were 6.6%, 15.8%, 19.1% and 31.1%, respectively (*p* < 0.01). Moreover, among women, the prevalence of cardiovascular diseases was 20.7% (*n* = 133) while the prevalence of endocrine diseases was 24.0% (*n* = 154). Between men, the corresponding percentages of cardiovascular and endocrine diseases were 30.2% (*n* = 150) and 8.5% (*n* = 42), respectively (both *p* < 0.01). 

We found statistically significant differences among the five geographical areas only among the cardiovascular disease groups (*p* = 0.03), whereas the highest percentage was reported in Ammochostos (30.0%) and the lowest in Paphos (15.9%). We did not find any statistically significant associations between cardiovascular (*p* = 0.11) and endocrine (*p* = 0.73) disease groups among residents of urban and rural regions. In addition, the largest prevalence of cardiovascular and endocrine diseases was identified among those who completed only primary education (68.2% and 34.8%, respectively) (*p* < 0.01). The prevalence of both diseases decreases as the educational level increases. Moreover, the largest prevalence of cardiovascular and endocrine diseases was reported among retired individuals and state employees (69.0% and 28.0% (*p* < 0.01) vs. 33.3% and 17.9%, 14% (*p* < 0.01), respectively) (Table 1).

### 3.3. Lifestyle Factors

We reported that 35% of the participants were current smokers (Table 2) while among them, 58% were men. We did not find any statistically significant association between smoking status and either cardiovascular or endocrine diseases groups (*p* < 0.05). However, we observed a higher proportion of current smokers among individuals with cardiovascular diseases (*n* = 112, 27.9%) and a smaller proportion among individuals with endocrine diseases (*n* = 62, 15.4%). In addition, we detected that more than 50% of the participants were physically inactive (*n* = 591), while significant differences in the prevalence of cardiovascular and endocrine diseases between the physical activity levels were observed (*p* < 0.01). Specifically, the prevalence of cardiovascular and endocrine diseases among not adequately physically active participants were 30.1% (*n* = 178) and 20.3% (*n* = 120), respectively, while the corresponding percentages among physically active participants were 19.6% (*n* = 108) and 13.8% (*n* = 76) (Table 2). The most common types of exercise were the gym (37.3%), jogging (11.2%), and walking/gait (11.2%). The largest percentages of individuals with both cardiovascular and endocrine diseases were identified in those who exercised less than one hour per week (32.8% and 22.4%, respectively, *p* < 0.01).

The median MedDietScore was 15/55 (q_1_ = 13, q_3_ = 18), which indicates that the diet of the Cypriot population is away from the traditional Mediterranean diet pattern (Table 2). We did not find any statistical difference between the Mediterranean diet adherence groups and either cardiovascular or endocrine disease groups. We found that participants consumed olive oil daily while the median value for the consumption of alcohol was 1, which indicates no or rare consumption among the participants. Individuals with cardiovascular and endocrine diseases consumed less meat and meat products compared to individuals without cardiovascular and endocrine diseases. In addition, 28.5% (*n* = 183) and 20.2% (*n* = 130) of the participants who reported no alcohol consumption and 20.0% (*n* = 11) and 10.9% (*n* = 6) of the participants who reported consuming alcohol daily were individuals with cardiovascular and endocrine diseases, respectively.

The median quality of sleep score showed that participants had a good quality of sleep, with 61% (*n* = 695) of them being in the good quality of sleep group and 39% (*n* = 445) being in the poor quality of sleep group (Table 2). We found significant differences in the quality of sleep between individuals with cardiovascular and endocrine diseases versus those without cardiovascular diseases and endocrine diseases. Specifically, in the poor quality of sleep group, we reported a higher prevalence of cardiovascular and endocrine diseases compared to the corresponding percentages in the good quality of sleep group (30.3% vs. 21.3% and 20.5% vs. 15.1%, respectively) (*p* < 0.01) (Table 2). 

We also reported a significantly higher BMI in individuals with cardiovascular and endocrine diseases compared to the individuals without cardiovascular and endocrine diseases (26.7 vs. 24.4 kg/m^2^ and 26.2 vs. 24.7 kg/m^2^, respectively) (*p* < 0.01) (Table 2). In the overweight and obese groups, we reported a higher prevalence of cardiovascular (*p* < 0.01) and endocrine diseases (*p* = 0.01) as compared with the corresponding percentages in the normal and underweight groups.

### 3.4. Profile of Cardiovascular and Endocrine Individuals

Table 3 presents the results of a hierarchical discriminant analysis providing the standardized Fisher’s classification function coefficients. The analysis suggests that individuals with cardiovascular and endocrine diseases had poorer quality of sleep, physical inactivity, and higher BMIs, which contribute to their classification as having the diseases (*p* < 0.01). When we applied the discriminant analysis separately by gender, the dominant factors remained the same for both men and women with regard to endocrine diseases (*p* < 0.01). However, for cardiovascular diseases, we reported that in men, smoking, not adequate physical activity, and a higher BMI are the dominant factors that seem to better characterize them.

The results by age group were not statistically significant for the 18–24 years old group. In the 25–44 years old group, we found statistically significant estimates for poorer quality of sleep, smoking, and a higher BMI for endocrine diseases (*p* < 0.01). Furthermore, in individuals aged 45–64 years old with cardiovascular diseases, the factors which seemed to better characterize them were a higher adherence to the Mediterranean diet, inadequate physical activity, and a higher BMI, while the corresponding factors in individuals with endocrine diseases were poorer sleep quality, inadequate physical activity, and a higher BMI. Finally, for individuals aged 65 years and older, we only found statistically significant estimates for those with endocrine diseases, with higher adherence to a Mediterranean diet, current smoking habits and physical activity, being the factors that contribute more to their categorization.

### 3.5. Combinations of Cardiovascular and Endocrine Diseases

**Figure 1:**
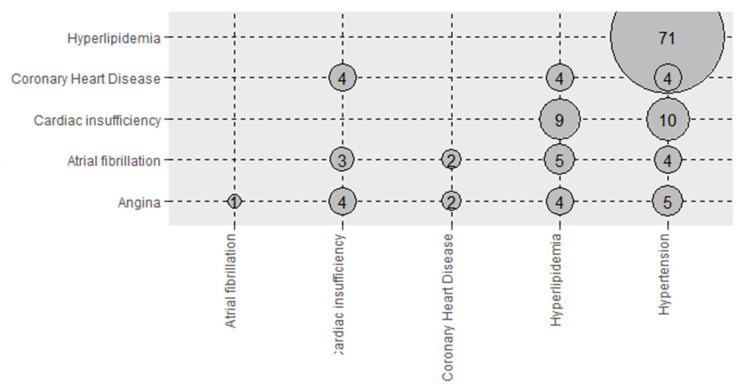
Combinations of cardiovascular diseases among people with at least two cardiovascular diseases.

**Figure 2:**
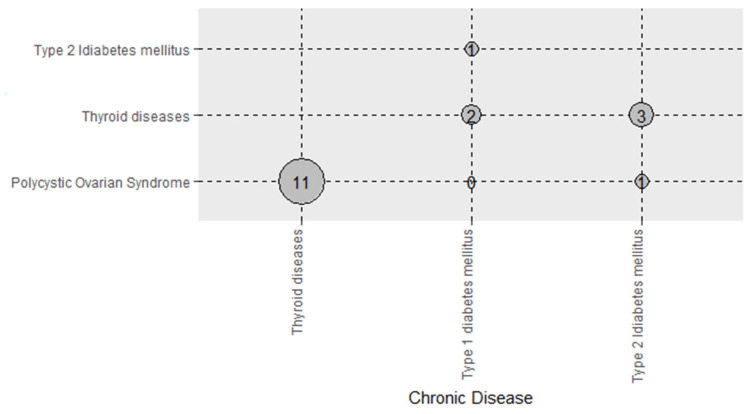
Combinations of endocrine diseases among people with at least two endocrine diseases.

The combinations of cardiovascular system diseases are presented in Figure 1. The most prevalent combination of the diseases of the cardiovascular system among people with at least two cardiovascular diseases was hypertension-hyperlipidemia (53.8%, *n* = 132). Furthermore, the most prevalent combination of the diseases of the endocrine system among participants with at least two endocrine diseases was thyroid diseases-polycystic ovarian syndrome (61.1%, *n* = 18) (Figure 2).

## 4. Discussion

To the best of our knowledge, this is the first study investigating the profile of individuals with cardiovascular and endocrine diseases in Cyprus using a large representative sample of the general adult population. The prevalence of cardiovascular diseases and endocrine diseases was 24.8% and 17.2%, respectively, with a higher prevalence of cardiovascular diseases in men, and a higher prevalence of endocrine diseases in women. The prevalence of cardiovascular and endocrine diseases was relatively high even among younger individuals. Moreover, the prevalence of smoking, physical activity, low adherence to the Mediterranean diet, poor quality of sleep and obesity among the study population was 35.5%, 48.0%, 32.9%, 39.0% and 13.6%, respectively. Our findings reveal that individuals with cardiovascular and endocrine diseases in Cyprus are characterized by a poorer quality of sleep, inadequate physical activity, and higher BMIs.

According to our findings, almost one-fourth of the adult population in Cyprus has at least one cardiovascular disease. This is in line with previous studies in the United Kingdom [35] and Greece [30], which reported a relatively high prevalence of such diseases. In addition, we found that the prevalence of cardiovascular diseases among participants aged 18–24 and 25–44 years old was 3.6% and 11.5%, respectively. Even though we did not accumulate information about congenital cardiovascular diseases, which could explain the high prevalence in younger individuals, our findings are consistent with previous studies [36,37]. In fact, it has been reported that the incidence of cardiovascular diseases has increased in adults aged 15–49 years old from 1990 to 2017 globally [36], especially in Western countries, probably due to a shift in lifestyle factors, such as unhealthy dietary habits and physical inactivity [37]. We reported that individuals who consumed less meat and meat products were more prone to CVD and endocrine diseases, however, it is not clear if the lower consumption of meat and meat products is a consequence of the presence of CVD and endocrine diseases. It has been reported that the Mediterranean diet decreases the risk of cardiovascular diseases [32,38], diabetes [39] and other metabolic morbidities [40] and this may play a role in the lower consumption of meat and meat products among individuals with cardiovascular and endocrine diseases.

Furthermore, we found a higher prevalence of cardiovascular diseases among men, which is in agreement with other epidemiological studies [41,42]. Our finding can be explained by the fact that men develop congenital heart defects at a younger age compared to women; they often smoke more and are more likely to have high blood pressure [43]. In fact, the WHO reported that about 40% of men and 9% of women smoke globally [44], and that 1 in 4 men and 1 in 5 women per a billion people have high blood pressure [44]. Moreover, the possible differences in cardiovascular diseases among men and women could be explained, since men are exposed to more environmental or lifestyle factors [45]. Likewise, sex steroids and their receptors can contribute to gender differences in the prevalence of cardiovascular diseases as they interact with other proteins and genes that have a role in cardiovascular disease pathogenesis [46]. 

The prevalence of endocrine diseases among the adult population of Cyprus was almost 18%. The relatively high prevalence estimates of endocrine disorders are also reported in other research studies [47,48,49]. In our study, women reported having three times the prevalence of endocrine diseases than men. It is known that there are differences between men and women regarding endocrine disorders due to the different hormones involved [50]. For instance, estrogen is one such hormone that has a significant effect on women. Specifically, estrogen affects fuel metabolism by reducing fatty acid oxidation, which leads to an increase in body fat [50]. The increase in body fat may affect the increased fat mass in women compared to men.

We also found a higher prevalence of endocrine diseases among the elderly individuals (>65 years old) compared to participants aged 18–64 years old, which may be due to the fact that this population group has different physiology compared to younger individuals, leading to a higher prevalence of endocrine diseases in older ages [51]. For instance, elderly individuals have a lower cutoff level of serum testosterone, hypopituitarism, hypothyroidism, osteoporosis, diabetes mellitus, and adrenal insufficiency, whilst several types of hypogonadism are all the more frequent among older individuals [51].

We also found that participants with only a primary education had the highest prevalence of cardiovascular and endocrine diseases as compared with participants with secondary or higher education. This inverse association between education and the prevalence of cardiovascular diseases is in agreement with other research studies in Northeastern Spain [52] and in Finland, Ireland and France [53,54]. Socioeconomic differences in health-related factors, including smoking status, were described in several European countries, especially among younger men and women [55]. Moreover, several research studies have found that a lower educational level is associated with a high prevalence of hypertension [56,57,58], current smoking habits [57,59,60,61], and a high prevalence of cholesterol [62].

Our findings suggest that individuals with cardiovascular and endocrine diseases are characterized by a poorer quality of sleep, with individuals who get less sleep having a higher risk of developing cardiovascular and endocrine diseases, which is in line with previous research [23,24]. Several studies suggested that sleep duration is associated with the presence of other cardiovascular risk factors (i.e., obesity, high blood pressure) [63,64] and an increased risk for cardiovascular events [65]. In addition, it has been shown that poor sleep increases the risk associated with hypertension [25,26] and multimorbidity [27]. A prospective cohort study, which examined 60,586 adults aged more than 40 years old, found that short sleep duration and poor quality of sleep are associated with an increased risk of coronary heart disease [66]. Poor sleep could also affect the endocrine system [25,26] leading to diabetes [67,68]. Furthermore, recent studies have reported that an abnormal quality of sleep is associated with a higher risk of metabolic syndrome and type II diabetes mellitus [69] and that patients with uncontrolled diabetes and insulin users had a higher risk of poor quality of sleep [70]. 

In our study, individuals with cardiovascular and/or endocrine diseases were on average not physically active and had a higher BMI. It is known that physical activity is associated with a lower risk of both cardiovascular and endocrine diseases [21,71]. A higher level of physical activity is associated with a reduced risk of type II diabetes mellitus and coronary heart disease and, more specifically, it has been reported that physical inactivity is responsible for 7% and 6% of type II diabetes mellitus and coronary heart disease burden, respectively [72,73]. Moreover, obesity is a major risk factor for cardiovascular diseases (i.e. hypertension, coronary heart disease, atrial fibrillation, heart failure) [74,75], while a recent systematic review and meta-analysis found the prevalence of endocrine disorders to be significantly higher among patients with obesity [76]. Physical inactivity and obesity are among the top ten behavior-related risk factors for the burden of disease in developed countries [76], which is in agreement with our study. We also reported a higher percentage of individuals with cardiovascular and endocrine diseases among the participants who are physically active, which is in contrast to other studies [77,78,79]. However, it is not clear if physical activity is a consequence of the presence of cardiovascular or endocrine diseases in an individual. For instance, physical activity reduces the risk of cardiovascular disease mortality [80] even though is unclear if there is a dose-response association. Hence, it is possible that cardiovascular as well as endocrine individuals became physically active after the diagnosis. 

Smoking status was a dominant characteristic of men with cardiovascular diseases. This finding is in agreement with two large population-based cohort studies that investigated the lifestyle-related factors and 10-year cardiovascular disease risk among the Greek population in which smoking in men was reported as an independent predictor of cardiovascular events over the 10-year period [81]. The fact that this finding was observed primarily in men could be explained by the high prevalence of current smoking in men compared to women (47% vs. 26%) which is consistent with the country’s health profile in 2019 [15].

### Limitation and Strengths

There are some limitations in our study that should be considered when interpreting our findings. First, the cross-sectional design used means that only associations could be examined, but not causal relationships. Furthermore, the severity of the disease is not considered, and all chronic diseases included in the study were provided directly by the participants based on diagnosis by a physician. Face-to-face interviews for the medical history assessment could be influenced by social desirability bias, but this was mitigated by having trained field workers conduct the interviews. 

Notwithstanding these limitations, the study has several strengths, as this is a large population-based study using a representative sample of both men and women of all ages (18+) and geographical areas of Cyprus. Other strengths include the collection of detailed data using a questionnaire where most of its sections have been validated (PSQI, FFQ), whilst the questionnaire was also pilot tested using a sample of 10% of the total sample of the study. Furthermore, detailed data were collected, including information about the demographic and socioeconomic characteristics of the participants, the presence of 47 chronic conditions, on the participants’ dietary habits, including the consumption of 11 food groups (non-refined cereals, fruits, vegetables, legume, potatoes, fish, meat and meat products, poultry, full-fat dairy products, olive oil and alcohol intake), on the quality of sleep and the level of stress, as well as information about smoking and exercise, etc.

## 5. Conclusions

Adult individuals with cardiovascular and endocrine diseases have poorer sleep quality, insufficient physical activity, and a higher BMI. Our findings could be used to develop programs that focus on the positive effects of improved sleep quality, physical activity, and lower BMI in people with cardiovascular or endocrine diseases. In addition, our study highlights the need for programs and interventions targeting individuals at risk of developing cardiovascular and/or endocrine disorders. Physicians, psychologists and other healthcare professionals should educate, inform, and encourage people to live healthier lifestyles. Future research should investigate the beneficial effects of those factors in cardiovascular and endocrine diseases on life expectancy, disability-free living, and overall quality of life.

## Figures and Tables

**Table 1:** Demographics and socioeconomic characteristics overall, and by participants’ health status (cardiovascular diseases and endocrine diseases groups).

		Cardiovascular Diseases			Endocrine Diseases		
**Characteristics**	Overall (*n* = 1140)	No (*n* = 856)	Yes (*n* = 283)	*p*-Value	No (*n* = 944)	Yes (*n* = 196)	*p*-Value
**Age (years)**, Mean (SD)	40.8 ± 16.9	35.9 ± 14.1	55.4 ± 16.3	**<0.01** ^f^	39.5 ± 16.3	47.2 ± 18.1	**<0.01** ^f^
**Age group**, *n* ^a^ (%)							
18–24	167 (14.7)	161 (96.4)	6 (3.6)	**<0.01** ^g^	156 (93.4)	11 (6.6)	**<0.01** ^g^
25–44	524 (46.0)	464 (88.5)	60 (11.5)		441 (84.2)	83 (15.8)	
45–64	314 (27.5)	188 (59.9)	126 (40.1)		254 (80.9)	60 (19.1)	
65+	135 (11.8)	44 (32.6)	91 (67.4)		93 (68.9)	42 (31.1)	
**Sex**, *n* ^b^ (%)							
Women	642 (56.4)	509 (79.3)	133 (20.7)	**<0.01** ^g^	488 (76.0)	154 (24.0)	**<0.01** ^g^
Men	497 (43.6)	347 (69.8)	150 (30.2)		455 (91.5)	42 (8.5)	
**Geographical area**, *n* ^c^ (%)							
Nicosia	493 (43.3)	366 (74.2)	127 (25.8)	**0.03** ^g^	410 (83.2)	83 (16.8)	0.06 ^g^
Limassol	311 (27.3)	222 (71.4)	89 (28.6)		243 (78.1)	68 (21.9)	
Larnaca	171 (15.0)	137 (80.1)	34 (19.9)		149 (87.1)	22 (12.9)	
Paphos	113 (9.9)	95 (84.1)	18 (15.9)		99 (87.6)	14 (12.4)	
Ammochostos	50 (4.5)	35 (70.0)	15 (30.0)		41 (82.0)	9 (18.0)	
**Residency**, *n* ^d^ (%)							
Urban	864 (76.3)	639 (75.1)	225 (79.8)	0.11 ^g^	715 (76.3)	149 (76.0)	0.93 ^g^
Rural	269 (23.7)	212 (24.9)	57 (20.2)		222 (23.7)	47 (24.0)	
**Educational status**, *n* ^d^ (%)							
Primary education	66 (5.8)	21 (31.8)	45 (68.2)	**<0.01** ^g^	43 (65.2)	23 (34.8)	**<0.01** ^g^
Secondary education	338 (29.8)	240 (71.0)	98 (29.0)		282 (83.4)	56 (16.6)	
Higher education	729 (64.4)	591 (81.1)	138 (18.9)		613 (84.1)	116 (15.9)	
**Job status**, *n* ^e^ (%)							
Private employee	432 (39.9)	362 (83.8)	70 (16.2)	**<0.01** ^g^	369 (85.4)	63 (14.6)	**<0.01** ^g^
State employee	218 (20.1)	157 (72.0)	61 (28.0)		179 (82.1)	39 (17.9)	
Freelance	100 (9.2)	79 (79.0)	21 (21.0)		86 (86.0)	14 (14.0)	
Unemployed	205 (18.9)	175 (85.4)	30 (14.6)		178 (86.8)	27 (13.2)	
Retired	129 (11.9)	40 (31.0)	89 (69.0)		86 (66.7)	43 (33.3)	

Abbreviations: SD, Standard Deviation; ^a^ *n* = 1140; ^b^ *n* = 1139; ^c^ *n* = 1138; ^d^ *n* = 1133; ^e^ *n* = 1084; ^f^ Differences between groups were tested using *t*-test; ^g^ Differences between groups were tested using chi^2^ test. Bold values indicate statistically significant associations (*p* < 0.05).

**Table 2:** Lifestyle factors overall, by participants with cardiovascular as well as with endocrine diseases.

		Cardiovascular Diseases			Endocrine Diseases		
**Characteristics**	Overall (*n* = 1139)	No (*n* = 856)	Yes (*n* = 283)	*p*-Value	No (*n* = 944)	Yes (*n* = 196)	*p*-Value
**Smoking habits**							
**Age of starting smoking ^a^**, Mean (SD)	18.7 ± 4.6	18.6 ± 4.1	19.1 ± 5.6	0.31 ^i^	18.8 ± 4.8	18.3 ± 3.4	0.41 ^i^
**Smoking status**, *n* ^a^ (%)							
Non-smoker	731 (64.5)	561 (76.7)	170 (23.3)	0.09 ^j^	598 (81.8)	133 (18.2)	0.24 ^j^
Current smoker	402 (35.5)	290 (72.1)	112 (27.9)		340 (84.6)	62 (15.4)	
**Physical activity level**							
**Physical activity**, *n* ^b^ (%)							
Not adequately physical active	591 (52.0)	413 (69.9)	178 (30.1)	**<0.01** ^j^	471 (79.7)	120 (20.3)	**<0.01** ^j^
Physical active	545 (48.0)	438 (80.4)	108 (19.6)		470 (86.2)	76 (13.8)	
**Type of exercise**, *n* ^c^ (%)							
Gym	146 (26.8)	127 (87.0)	19 (13.0)	**<0.01** ^j^	127 (87.0)	19 (13.0)	0.58 ^j^
Combination	189 (34.7)	156 (82.5)	33 (17.5)		164 (86.8)	25 (13.2)	
Walking/Gait	62 (11.4)	36 (58.1)	26 (41.9)		48 (77.4)	14 (22.6)	
Jogging	27 (5.0)	22 (81.5)	5 (18.5)		26 (96.3)	1 (3.7)	
Swimming	22 (4.0)	16 (72.7)	6 (27.3)		18 (81.8)	4 (18.2)	
Football	21 (3.9)	14 (66.7)	7 (33.3)		20 (95.2)	1 (4.8)	
Pilates/Yoga	18 (3.3)	13 (72.2)	5 (27.8)		14 (77.8)	4 (22.2)	
Dance/Zumba	10 (1.8)	10 (100.0)	0 (0.0)		9 (90.0)	1 (10.0)	
Martial arts	10 (1.8)	8 (80.0)	2 (20.0)		9 (90.0)	1 (10.0)	
Cycling	10 (1.8)	9 (90.0)	1 (10.0)		9 (90.0)	1 (10.0)	
Basketball	8 (1.5)	6 (75.0)	2 (25.0)		6 (75.0)	2 (25.0)	
Volleyball	5 (0.9)	5 (100.0)	0 (0.0)		5 (100.0)	0 (0.0)	
Cross fit/TRX	5 (0.9)	5 (100.0)	0 (0.0)		4 (80.0)	1 (20.0)	
Handball	5 (0.9)	5 (100.0)	0 (0.0)		5 (100.0)	0 (0.0)	
Other ^d^	7 (1.3)	5 (71.4)	2 (28.6)		6 (85.7)	1 (14.3)	
**Hours of exercise per week**, *n* ^e^ (%)							
Less than 1 h	58 (11.3)	39 (67.2)	19 (32.8)	**<0.01** ^j^	45 (77.6)	13 (22.4)	**0.17** ^j^
1–3 h	208 (39.3)	169 (81.2)	39 (18.8)		178 (85.6)	30 (14.4)	
3–6 h	151 (28.5)	121 (80.1)	30 (19.9)		131 (86.7)	20 (13.3)	
6–9 h	77 (14.6)	70 (90.9)	7 (9.1)		70 (90.9)	7 (9.1)	
More than 9 h	33 (6.3)	26 (78.8)	7 (21.2)		31 (93.9)	2 (6.1)	
**Dietary habits**							
**MedDietScore** (range: 0–55), Median (IQR)							
**MedDietScore**	15 (13, 18)	15 (13, 18)	16 (13, 18)	0.91 ^k^	16 (13, 18)	15 (13, 18)	0.30 ^k^
**Mediterranean Diet adherence**, *n* ^f^ (%)							
Low (≤13)	370 (32.9)	286 (77.3)	84 (22.7)	0.204 ^j^	298 (80.5)	72 (19.5)	0.381 ^j^
Moderate (14–18)	511 (45.4)	372 (72.8)	139 (27.2)		429 (84.0)	82 (16.0)	
High (≥19)	245 (21.7)	190 (77.5)	55 (22.5)		205 (83.7)	40 (16.3)	
**Food consumption** (0: no consumption—5: daily), Median (IQR)							
Full-fat dairy products	2.5 (1.5, 3)	2.5 (1.5, 3)	2.5 (1.5, 3)	0.95 ^k^	2.5 (1.5, 3)	2.5 (1.5, 3)	0.88 ^k^
Non-refined cereals	2 (1.3, 2.3)	2 (1.3, 2.3)	1.7 (1, 2.3)	0.16 ^k^	2 (1.3, 2.3)	2 (1.3, 2.3)	0.74 ^k^
Meat and meat products	2.5 (2.2, 3.2)	2.7 (2.2, 3.2)	2.2 (2, 3)	**<0.01** ^k^	2.7 (2.2, 3.2)	2.5 (2, 3)	**0.03** ^k^
Poultry	3 (2, 4)	3 (2, 4)	3 (2, 3)	**0.01** ^k^	3 (2, 4)	3 (2, 3)	0.58 ^k^
Fish	2 (1.5, 2)	2 (1.5, 2)	2 (1.5, 2.5)	**0.04** ^k^	2 (1.5, 2)	2 (1.5, 2)	0.80 ^k^
Vegetables	3 (2.2, 3.5)	3 (2.2, 3.5)	3 (2.7, 3.7)	0.17 ^k^	3 (2.2, 3.5)	3 (2.2, 3.5)	1.0 ^k^
Potatoes	3 (2.3)	3 (2.3)	3 (2.3)	0.97 ^k^	3 (2.3)	3 (2.3)	0.99 ^k^
Fruits	2.8 (2, 3.6)	2.8 (2, 3.6)	3 (2.2, 3.8)	0.06 ^k^	2.8 (2, 3.6)	3 (2.25, 3.6)	0.59 ^k^
Legumes	3 (2.3)	3 (2.3)	3 (3, 4)	0.23 ^k^	3 (2, 3)	3 (3, 3)	0.99 ^k^
Olive oil	4 (3, 5)	4 (2, 5)	4 (3, 5)	**<0.01** ^k^	4 (3, 5)	4 (2, 5)	0.28 ^k^
Alcohol intake	1 (1, 2)	1 (1, 2)	1 (1, 2)	**<0.01** ^k^	1 (1, 2)	1 (1, 2)	**0.02** ^k^
**Quality of sleep**							
**Quality of sleep score** (range: 0–21), Median (IQR)							
**Quality of sleep score**	5 (3, 7)	5 (3, 7)	5 (3, 8)	**<0.01** ^k^	5 (3, 7)	5 (3, 8)	**0.01** ^k^
**Quality of sleep**, *n* ^g^ (%)							
Good (≤5)	695 (61.0)	547 (78.7)	148 (21.3)	**<0.01** ^j^	590 (84.9)	105 (15.1)	**0.02** ^j^
Poor (>5)	445 (39.0)	310 (69.7)	135 (30.3)		354 (79.5)	91 (20.5)	
**Hours of sleeping**, Mean (SD)	6.6 ± 1.4	6.6 ± 1.4	6.3 ± 1.4	**<0.01** ^i^	6.6 ± 1.4	6.4 ± 1.5	0.19 ^i^
**Minutes to fall asleep**, Mean (SD)	20.3 ± 23.8	19.9 ± 23.8	21.8 ± 23.7	0.24 ^i^	19.6 ± 22.9	23.9 ± 27.3	**0.02** ^i^
**Obesity**							
**BMI (kg/m^2^)**, Mean (SD)	25.0 ± 4.6	24.4 ± 14.7	26.7 ± 4.3	**<0.01** ^i^	24.7 ± 4.4	26.2 ± 5.5	**<0.01** ^i^
**BMI**, *n* ^h^ (%)							
Normal	565 (50.4)	467 (82.6)	98 (17.4)	**<0.01** ^j^	486 (86.0)	79 (14.0)	**0.01** ^j^
Underweight	42 (3.7)	41 (97.6)	1 (2.4)		36 (85.7)	6 (14.3)	
Overweight	362 (32.3)	241 (66.6)	121 (33.4)		290 (80.1)	72 (19.9)	
Obese	152 (13.6)	94 (61.8)	58 (38.2)		116 (76.3)	36 (23.7)	

Abbreviations: SD, Standard Deviation; IQR, Interquartile Range; ^a^ *n* = 1133; ^b^ *n* = 1136; ^c^ *n* = 545; ^d^ Aerobic, Tennis, Track, Surf/Wakeboard; ^e^ *n* = 527; ^f^ *n* = 1126; ^g^ *n* = 1140; ^h^ *n* =1140; ^i^ Differences between groups were tested using *t*-test; ^j^ Differences between groups were tested using chi^2^ test. ^k^ Differences between groups were tested using Wilcoxon rank-sum test. Both values indicate statistically significant associations (*p* < 0.05).

**Table 3:** Hierarchical discriminant analysis (standardized Fisher’s classification function coefficients, reference category: No cardiovascular diseases and No endocrine diseases, respectively).

	Overall	Gender		Age Group			
		Women	Men	18–24	25–44	45–64	65+
**Cardiovascular diseases**							
Mediterranean diet score (per 1 unit)	0.19	0.31	0.02	0.07	0.12	**0.44**	**0.24**
Quality of sleep score (per 1 unit)	**0.37**	**0.47**	0.17	**0.54**	**0.45**	0.22	0.14
Current smoker (Yes, No)	0.12	0.10	**0.18**	0.11	0.21	0.09	0.11
Physically active (Yes, No)	**−0.36**	**−0.39**	**−0.44**	**−0.33**	**−0.33**	**−0.39**	**−0.50**
BMI (per 1 kg/m^2^)	**0.78**	**0.67**	**0.92**	**0.73**	**0.75**	**0.79**	**0.72**
**Endocrine diseases**							
Mediterranean Diet score (per 1 unit)	−0.13	0.39	0.08	−0.07	−0.30	0.19	**0.43**
Quality of sleep score (per 1 unit)	**0.58**	**−0.41**	**0.71**	**0.69**	**0.57**	**0.51**	−0.33
Current smoker (Yes, No)	−0.28	0.34	−0.21	**−0.43**	**−0.25**	−0.08	**0.56**
Physically active (Yes, No)	**−0.34**	**0.41**	**−0.23**	−0.30	−0.21	**−0.27**	**0.67**
BMI (per 1 kg/m^2^)	**0.63**	**−0.56**	**0.64**	**0.38**	**0.71**	**0.73**	−0.20

Bold values indicate the most dominant factors in each classification.

## Data Availability

The datasets used and/or analyzed during the current study are available from the corresponding author on reasonable request.
